# Impact of HepG2 Cells Glutathione Depletion on Neutral Sphingomyelinases mRNA Levels and Activity

**DOI:** 10.3390/cimb45060318

**Published:** 2023-06-08

**Authors:** Marie Gamal, Hatem Tallima, Hassan M. E. Azzazy, Anwar Abdelnaser

**Affiliations:** 1 Department of Chemistry, School of Sciences and Engineering, The American University in Cairo, New Cairo 11835, Egypt; 2 Institute of Global Health and Human Ecology, School of Sciences and Engineering, The American University in Cairo, New Cairo 11835, Egypt

**Keywords:** HCC, HepG2 cells, sphingomyelin, sphingomyelinase, glutathione, ROS, BSO, ceramide

## Abstract

Liver cancer is a prevalent form of cancer worldwide. While research has shown that increasing sphingomyelin (SM) hydrolysis by activating the cell surface membrane-associated neutral sphingomyelinase 2 (nSMase2) can control cell proliferation and apoptosis, the role of total glutathione depletion in inducing tumor cell apoptosis via nSMase2 activation is still under investigation. Conversely, glutathione-mediated inhibition of reactive oxygen species (ROS) accumulation is necessary for the enzymatic activity of nSMase1 and nSMase3, increased ceramide levels, and cell apoptosis. This study evaluated the effects of depleting total glutathione in HepG2 cells using buthionine sulfoximine (BSO). The study assessed nSMases RNA levels and activities, intracellular ceramide levels, and cell proliferation using RT-qPCR, Amplex red neutral sphingomyelinase fluorescence assay, and colorimetric assays, respectively. The results indicated a lack of nSMase2 mRNA expression in treated and untreated HepG2 cells. Depletion of total glutathione resulted in a significant increase in mRNA levels but a dramatic reduction in the enzymatic activity of nSMase1 and nSMase3, a rise in ROS levels, a decrease in intracellular levels of ceramide, and an increase in cell proliferation. These findings suggest that total glutathione depletion may exacerbate liver cancer (HCC) and not support using total glutathione-depleting agents in HCC management. It is important to note that these results are limited to HepG2 cells, and further studies are necessary to determine if these effects will also occur in other cell lines. Additional research is necessary to explore the role of total glutathione depletion in inducing tumor cell apoptosis.

## 1. Introduction

The incidence of hepatocellular carcinoma (HCC) dramatically increased over the past two decades and is presently the fourth most common cause of cancer-related death worldwide [1,2]. Multiple etiologies can lead to HCC, viral as hepatitis B and C, or non-viral such as obesity, diabetes, non-alcoholic steatohepatitis, and dietary exposures, including aflatoxins [1,2,3]. HCC treatment can be either surgical via liver lobes excision or transplantation. Still, patients and liver donors often drop out of candidacy or non-surgical methods, including radiotherapy, chemotherapy, biotherapy, and hormonal therapy [1,2,3,4,5,6].

Evidence is now mounting for the role of cell surface membrane sphingomyelin (SM) in tumor initiation, immune escape, growth, and metastasis [7]. Notably, inordinate SM amounts were detected on the surface membrane of HepG2 and Huh-7 HCC cell lines and tissues and correlated with malignancy [7,8]. Sphingomyelin content was upregulated in HCC tumor tissue compared to matched normal liver tissue from each of the 46 patients [9]. Overexpression of sphingomyelin synthase 1 promoted HepG2 cell growth and migration [10]. The gene encoding SM phosphodiesterase 3 (SMPD3), also referred to as neutral sphingomyelinase 2 (nSMase2), which is predominantly localized at the cytosolic leaflet of the plasma membrane, and responsible for surface membrane SM hydrolysis was identified as a tumor suppressor gene in primary HCC [11]. Gene overexpression elicited diminished cellular proliferation, and knockdown promoted tumor invasiveness and migratory capacities. In contrast, low levels of the expressed enzyme were associated with HCC aggressiveness and early recurrence after surgery [10]. Notably, *nSMase2* gene hypermethylation and loss of expression were detected in the majority of the examined primary HCC [12]. The HCC cell lines JHH-7 and HepG2 displayed low nSMase-2 protein levels [11,12]. Increased liver tumor formation was recorded in neutral sphingomyelinase-2-deficient mice [13]. Other Mg^++^-dependent nSMases, notably nSMase1 (SMPD2) and nSMase3 (SMPD4), also catalyze the cleavage of the phosphodiester bond in SM to ceramide and phosphocholine, but unlike nSMase2, are not associated with the cell surface membrane, are located in the endoplasmic reticulum, Golgi apparatus, and nuclear matrix of multiple tissues [14,15,16]. Controversial results were recorded regarding the expression of nSMase1, which was significantly downregulated [17] or considerably overexpressed [18] in HCC compared to normal para-carcinoma tissues.

The nSMase1 enzymatic activity requires reducing agents and is reversibly inhibited by reactive oxygen species (ROS) and oxidized glutathione (GSSG) but irreversibly inhibited by reactive nitrogen species such as peroxynitrite [19,20]. Conversely, reduced glutathione (GSH) was reported to considerably inactivate nSMase2, with its increased enzymatic activity during aging caused by a 60–70% decrease in hepatocyte glutathione levels [21]. Indeed, the levels of glutathione, the major intracellular antioxidant of the body, decline with aging, resulting in oxidative stress in old humans and rodents and senescent cells in culture [22]. In liver cells, the low nSMase2 activity is regulated by antioxidants and can be activated during oxidative stress and glutathione depletion [22,23] (Figure S1). In support, hydrogen peroxide-induced activation of nSMase2, ceramide generation, and apoptosis, which was inhibited by glutathione [14,24]. Several studies have demonstrated that oxidative stress leads to a marked increase in nSMase2 activity, documenting glutathione as an endogenous nSMase2 inhibitor. Hence, intracellular glutathione depletion has been proposed as a mechanism for nSMase2 activation [21,22,23,24,25], especially since elevated glutathione levels in tumor cells were associated with tumor progression and increased resistance to chemotherapeutic drugs [26,27,28].

To resolve this controversy, we investigated the specific role of total glutathione in HepG2 cells survival via its depletion with its biosynthesis inhibitor, buthionine sulfoximine (BSO) [29] (Figure S2), and assessing the resultant effects on ROS generation, nSMases 1, 2, and 3 mRNA levels and activity, ceramide content, and cell proliferation.

## 2. Materials and Methods

### 2.1. Materials

The human hepatoma HepG2 cell line was obtained from ATCC (HB-8065), (Manassas, VA, USA) in Dulbecco’s Modified Eagle Medium, high glucose DMEM supplemented with 10% fetal bovine serum was purchased from Gibco™ (Waltham, MA, USA). Amplex red sphingomyelinase assay kit (A12220), dimethyl sulfoxide (DMSO) (67-68-5), chloroform (HPLC grade; C607SK-1), isopropanol (HPLC grade; BP26324), ethanol (HPLC grade; 64-17-5), revertaid cDNA kit (K1621), maxima SYBR green qPCR (K0251), mRNA primers (designed by NCBI primer blast tool), BCA assay kit (A53225, A53226, and A53227), were all purchased from Thermo Fisher Scientific (Waltham, MA, USA). Penicillin-streptomycin mixture, 100× (09-757F), and phosphate-buffered saline (10×) (PBS) (17-516Q), trypsin (CC-5012), 3-(4, 5-dimethylthiazol-2-yl)-2, 5- diphenyltetrazolium bromide (MTT; 298-93-1), H_2_DCFDA dye (5935), were purchased from Tocris Bioscience (Bristol, UK). DMEM (with 4.5 g/L glucose, without L-glutamine, without phenol red, 12-917F) was purchased from Lonza-Bioscience (Basel, Switzerland). QiAzol lysis buffer (79306) and RNAse/DNAse free water (129114) were purchased from Qiagen (Hilden, Germany). Ethylenediamine tetraacetic acid (39760.01) and Tris-HCl (37192.01) were purchased from Serva (Perm Krai, Russia). L-BSO 97% TLC grade (B2515), quantification kit for oxidized and reduced glutathione (38185), cambinol ≥97% (HPLC) (C0494), myriocin ≥98% (HPLC) (M1177), hydrochloric acid (szbe1990v) was purchased from Merk (Darmstadt, Germany). The ceramide kit (MBS7254089) was purchased from MyBioSource, (San Diego, CA, USA). 5-sulphosalicylic acid dihydrate extra pure (S-08755) was purchased from Oxford Lab Fine Chem LLP (Maharashtra, India). Protease inhibitor cocktail (100×) (5871) was purchased from Cell Signaling Technology (Danvers, MA, USA).

### 2.2. Cell Cultures

HepG2 cells were grown in 75 cm^2^ flasks, using DMEM containing 10% FBS and 1% penicillin-streptomycin mixture (complete medium) until 80% confluence at 37 °C and 5% CO_2_ humidified incubator. The old medium was discarded, and the cells were washed with 5 mL of 1× PBS. Then, the PBS was discarded, and 4 mL of 0.025% trypsin in PBS was added. The flask was shaken for 1 min, trypsin was discarded, and the cells were incubated for 4 min in a 37 °C and 5% CO_2_ humidified incubator. In the flask, 4 mL of DMEM/10% FBS were added and pipetted up and down for 1 min to ensure that all the cells were evenly suspended before seeding in different plate formats based on the experiment performed.

### 2.3. BSO Treatment and Total Glutathione Detection Assay

HepG2 cells were seeded in 75 cm^2^ tissue culture flasks or 96-well plates (5 × 10^3^ HepG2 cells/well) and left to incubate at 37 °C and 5% CO_2_ humidified atmosphere for 24 h. Cells were then exposed for 24 h to BSO at 0.00, 0.05, 0.1, 0.5, 1, 5, 10, 50, and 100 µM in a complete medium. The media were discarded, the cells were washed with PBS, which was discarded, and cells were collected via trypsin treatment and washed with PBS. The supernatant was discarded, and 80 μL of 10 mM HCl was added. Cells were lysed by freezing and thawing twice, then 20 μL of 5% sulfosalicylic acid dihydrate (SSA) was added. The mixture was centrifuged at 8000× *g* for 10 min. Finally, the supernatant was transferred to a new tube, and deionized water was added to reduce the SSA concentration to 0.5% for the assay. Ten μL of the cell lysates were added to each well in a 96-well plate; then the assay was performed according to the manufacturer’s instructions. Briefly, 30 μL of 5% SSA and 120 μL buffer were added to each well. After one h incubation period at 37 °C, 20 μL each of substrate, coenzyme, and enzyme working solutions were added to each well. Finally, the plate was incubated for 10 min at 37 °C and 5% CO_2_. The absorbance was read immediately at 405 nm. The total glutathione content was calculated using a standard curve according to the manufacturer’s instructions [22,30]. 

### 2.4. Reactive Oxygen Species Assay

To determine the degree of ROS generation induced by the BSO treatment on HepG2 cells, a fluorometric assay was used, utilizing the unique intracellular oxidation of 2′,7′-dichlorofluorescein diacetate (DCF-DA) [31,32]. Cells were grown for 24 h in a 96-well plate and then pretreated with five μM H_2_DCFDA for two h (in 100 μL medium) before the addition of 0.1, 0.5, and 1 μM BSO (in another 100 μL), a total of 200 μL. Pilot studies indicated a lack of need for higher BSO concentrations to achieve highly significant (*p* < 0.0001) increases in ROS levels. The plate was incubated for 24 h in the dark at 37 °C and 5% CO_2_. Then, fluorescence was measured at excitation and emission wavelengths of 485 and 535 nm, respectively. The change in ROS was calculated as a percentage of the control where the control is set at 100%. The ROS level was calculated using the following equation: ROS level (%) = (A sample − A blank/A control − A blank) × 100.

### 2.5. Isolation of Total RNA, Quantitative Reverse Transcription, and Real-Time PCR

After the 6 h or 24 h incubation period with BSO, total RNA was extracted using QIAzol lysis reagent according to the manufacturer’s instructions. The aqueous phase containing RNA was transferred to a new micro-centrifuge tube. After that, 150 µL isopropanol were added, incubated for 10 min, and then centrifuged for another 10 min at 12,000× *g* at 4 °C. The precipitated pellet was washed with 70% ethanol, vortexed briefly, and then centrifuged at 7500× *g* at 4 °C for 5 min. The supernatant was discarded, and the micro-centrifuge tubes were left to air dry for 5–10 min. Finally, the pellet was resuspended in 25 µL nuclease-free water [33]. The RNA samples were quantified by measuring their absorbance at 260 nm (ng/µL), using NanoDrop Spectrophotometer (Thermo Fisher Scientific), and were stored at −80 °C until use.

The RNA first-strand complementary DNA (cDNA) was synthesized using the Revertaid cDNA synthesis kit according to the manufacturer’s guidelines. Briefly, 1 μg of the total RNA of each sample was diluted with nuclease-free water up to 10 µL. Then 10 µL from the cDNA reaction was added to each sample for a final volume reaction of 20 µL. The cDNA reaction master mix is composed of 4 μL 5× reverse transcription (RT) buffer, 2 μL 10 mM dNTP mix (100 mM), 1 μL RT random Hexamer primers, and 1 μL Oligo (dt)18 primer, 1 μL RevertAid M-MuLV RT (200 U/μL) reverse transcriptase, 1 μL RiboLock RNase Inhibitor (20 U/µL). The final reaction mixture was subjected to the following thermal cycle conditions: 25 °C for 5 min, followed by 42 °C for 60 min, and then 70 °C for 5 min to terminate the reaction, and finally cooled to 4 °C in 96 well Thermal cycler (Applied Biosystems, Foster City, CA, USA). The completed reaction was stored at −80 °C until further analysis. Before real-time polymerase chain reaction (qPCR) quantification, the newly synthesized cDNA was diluted 1:3 with nuclease-free water by adding 40 µL nuclease-free water to the 20 µL cDNA, from which 3 µL were taken for the real-time polymerase chain reaction (PCR) [33].

The primers used for real-time PCR were generated using the online NCBI primer designing tool (https://www.ncbi.nlm.nih.gov/tools/primer-blast/, accessed on 27 March 2023) and synthesized at Thermo Fischer (Table 1). The SYBR green mRNA reaction was of total volume 12.5 μL which contain: 0.375 μL of 10 μM forward primer and 0.375 μL of 10 μM reverse primer (equivalent to a final primer concentration of 0.3 μM), 6.25 μL of SYBR Green Universal Mastermix, 2.5 μL of nuclease-free water, and 3 μL of cDNA sample (equivalent to 50 ng cDNA). The conditions for the amplification reactions of mRNA were as follows: 10 min at 95 °C and 40 cycles of 95 °C for 15 s and 60 °C for 1 min [22,33]. The fold change in the target genes between treated and untreated cells, normalized by the level of GAPDH was determined using the equation fold change = 2^−Δ(ΔCt)^, where ΔCt = Ct_(target)_ − Ct_(GAPDH)_ and Δ(ΔCt) = ΔCt_(treated)_ − ΔCt_(untreated)_.

### 2.6. Preparing Cell Lysates and Determination of Protein Content

In a 6-well plate, 25 × 10^5^ cells per well were seeded and incubated for 24 h at 37 °C and 5% CO_2_. Cells were treated for 24 h with 0.1, 0.5, 1, 5, and 10 µM of BSO, in parallel with 30 µM the sirtuin inhibitor cambinol, recently found to be an nSMase2 specific inhibitor [34], or ten µM myriocin, which prohibits the initial step of SM synthesis via inhibiting acetyl coenzyme A and L-serine condensation [35]. The concentrations used were selected based on preliminary viability studies. The cells were then washed and lysed using a lysis solution composed of 25 mM Tris-HCl, five mM EDTA, and 1X protease inhibitor cocktail, pH (7.4). According to the manufacturer kit procedure, protein content was evaluated using bicinchoninic acid (BCA) reagent. The reaction was read at an absorbance of 562 nm [36].

### 2.7. Amplex Red Sphingomyelinase Assay

The volume equivalent to 50 µg lysate protein was added in different wells in a 96-well plate and assessed using the Amplex red NEUTRAL sphingomyelinase assay, following the manufacturer’s instructions. Fluorescence was measured after 20 min of incubation at room temperature at an excitation of 540 nm and emission at 590 nm. The nSMase activity was calculated using the following equation: nSMase activity (%) = (A sample − A blank/A control − A blank) × 100. The change in nSMase activity was calculated as a percentage of the control where the control is set at 100% [37].

### 2.8. Ceramide Detection Assay

Cell lysate samples were diluted 1:1 with PBS. To the enzyme-linked immunosorbent assay strip wells (MyBioSource) coated with polyclonal anti-ceramide antibody, 100 μL of each sample was added, ten μL balance solution was dispensed, and 50 μL of the conjugate was added to each well. Then, the plate was covered and incubated for one h at 37 °C. After incubation, the plate was washed five times with the 1× wash solution. The plate was tap-dried, and 50 μL of substrate A and B were added to each well, followed by a 15 min incubation period at 37 °C. After the incubation period, 50 μL of stop solution was added, and the absorbance was measured at 450 nm. The ceramide content was calculated according to a standard curve [38].

### 2.9. Cell Proliferation

After BSO treatment and washing, HepG2 cells were incubated with the MTT solution. After that, 200 µL 99.5% isopropyl alcohol was added per well. After one h shaking, the absorbance was measured at 540 nm, and cell proliferative capacity was calculated according to the following equation: Cell proliferation (%) = (A sample − A blank/A control − A blank) × 100 [38,39].

### 2.10. Statistical Analysis

Data are presented as means ± SE for the indicated number of independently performed experiments. Two-way Analysis of Variance (ANOVA) and Student *t*-test was used to identify the statistical significance between multiple groups. A *p*-value < 0.05 was considered statistically significant. The comparative analysis of results between different experimental groups respective to their corresponding controls was conducted using Graph Pad Prism (Version 8; San Diego, CA, USA). 

## 3. Results

### 3.1. Effect of Different Concentrations of BSO on Total Glutathione Content in HepG2

**Figure 1:**
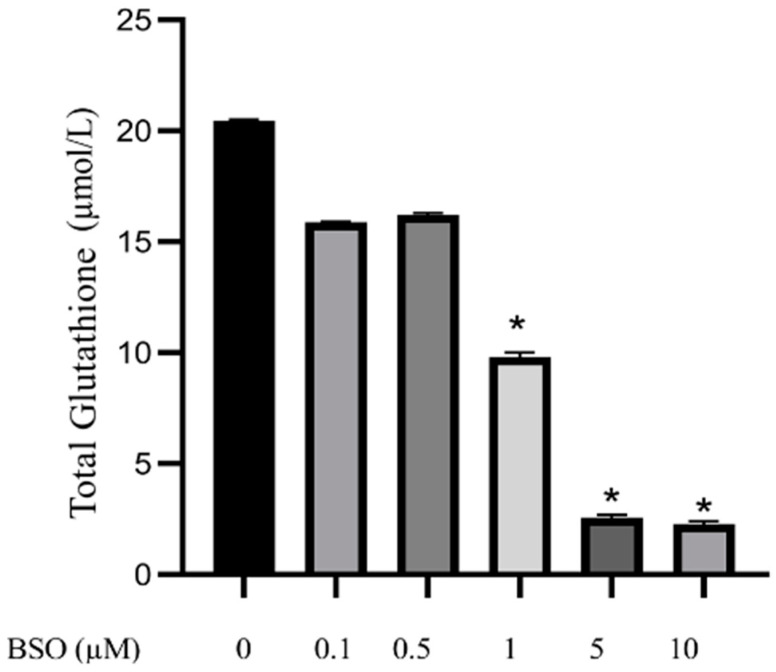
Effect of different BSO concentrations on total glutathione content in HepG2. HepG2 cells were treated with BSO for 24 h. Lysate total glutathione content was determined using a colorimetric assay. Data are expressed as the concentration of total glutathione (µmol/L) ± S.E. (n = 3). Comparisons are statistically analyzed by ANOVA and *t*-test and compared to the control (0 μM BSO). * *p* < 0.0001.

To determine the effect of BSO on the total glutathione content, HepG2 cells were treated with 0.1, 0.5, 1, 5 and 10 µM BSO for 24 h, and cell lysates prepared and protein content determined. Results of independent experiments showed that BSO at 1, 5 and 10 µM significantly (*p* < 0.0001) reduced the total glutathione content by 53, 88.1 and 89.5%, respectively, while BSO at 0.1, and 0.5 µM non significantly decreased total glutathione content by 22.6 and 21.2%, respectively, compared to vehicle treated control cells (Figure 1).

### 3.2. Effect of BSO on ROS Production in HepG2

**Figure 2:**
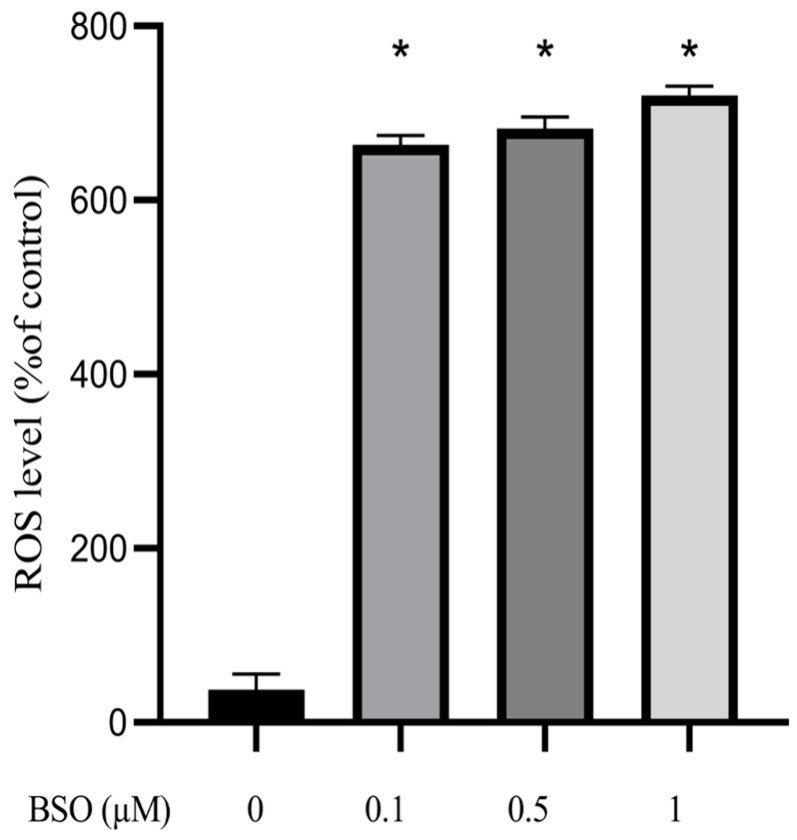
Effect of BSO on ROS production in HepG2. HepG2 cells were treated with BSO for 24 h. ROS activity was measured using a fluorometric assay of intracellular oxidation of DCFDA dye. Data are expressed as a percentage of control (0 μM BSO) ± S.E. (n = 8). * *p* < 0.0001.

The effect of different BSO concentrations on ROS production in HepG2 cells is shown in Figure 2, whereby BSO at 0.1, 0.5, and 1 µM concentrations was enough for significantly (*p* < 0.0001) increasing ROS production in HepG2 cells by 663.5, 682.6, and 721.7%, respectively, compared to 0 μM BSO-treated cells, hereafter referred to as control cells. A slight decrease in total glutathione content induced by BSO concentrations of 0.1 and 0.5 μM translated in such a ROS burst, indicating that cell glutathione thoroughly performs its anti-oxidative task provided its intracellular content is not impaired, as in aging [21,22].

### 3.3. Effect of BSO on nSMases mRNA Levels in HepG2

**Figure 3:**
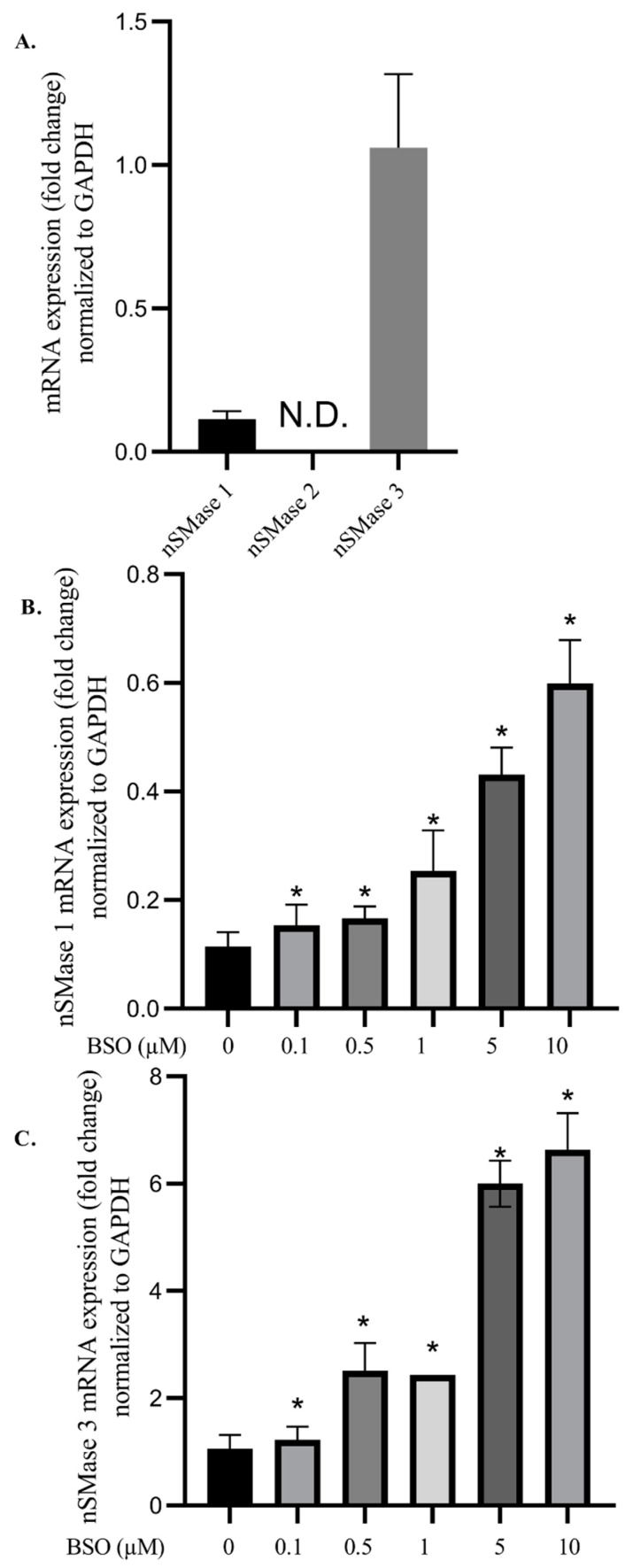
nSMase1, 2, and 3 mRNA content in HepG2. mRNA levels were measured using RT-qPCR and normalized to GAPDH (**A**). nSMase1 (**B**) and nSMase3 (**C**) mRNA levels in HepG2 lysates following cell treatment with BSO for 6 h. Data are expressed as mean ± S.E. (n = 3), and statistically analyzed by ANOVA and *t*-test compared to control lysates of control cells. *, *p* < 0.05; N.D., not detected.

The nSMase1, 2, and 3 mRNA expression levels in cell lysates were measured using RT-qPCR. Our results demonstrated that nSMase2 mRNA is not detectable (N.D.) in HepG2 cells before or after BSO treatment. nSMase1 RNA level was ten times higher than nSMase3 (Figure 3A). Furthermore, HepG2 cells treated with BSO at 0.1, 0.5, 1, 5, and 10 μM showed significantly (*p* < 0.005) increased nSMase1 mRNA levels by 134.5, 145.7, 222.4, 552.9, and 1502.8% respectively, compared to control cells (Figure 3B). Similarly, nSMase3 mRNA levels significantly (*p* < 0.05) increased in response to BSO at 0.1, 0.5, 1, 5, and 10 μM by115.3, 237, 324.1, 596.9 and 657%, respectively, compared to control cells (Figure 3C).

### 3.4. Effect of BSO, Cambinol, and Myriocin on nSMase Activity

**Figure 4:**
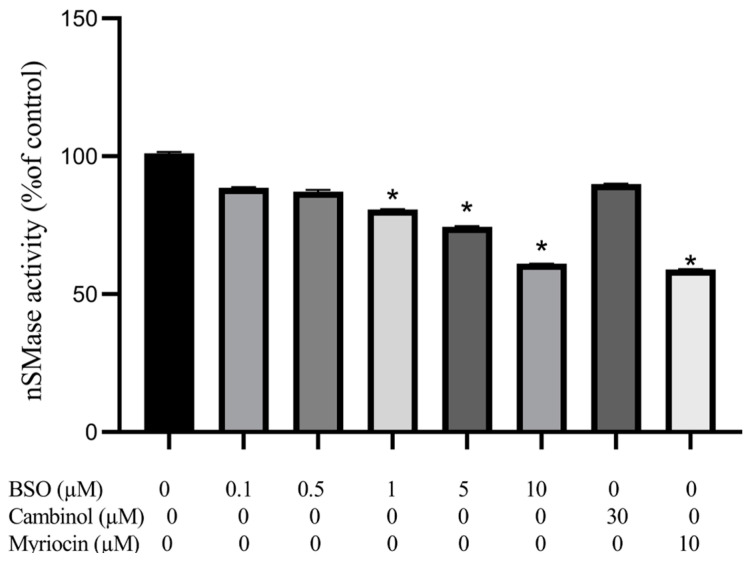
Effect of BSO, cambinol, and myriocin on HepG2 nSMase activity. HepG2 cells were exposed to BSO (0.1, 0.5, 1, 5 and 10 µM), cambinol (30 µM), or myriocin (10 µM) for 24 h and nSMase activity in cell lysates was measured using Amplex red sphingomyelinase fluorometric assay. Data are expressed as a percentage of control (at 100%) ± S.E. (n = 3). Data are statistically analyzed by ANOVA and *t*-test compared to control. *, *p* < 0.05.

HepG2 lysate was assessed for nSMase activity following cell treatment in parallel with BSO, the sirtuin inhibitor cambinol, recently found to be a nSMase2 inhibitor [34] or myriocin, which prohibits the initial step of SM synthesis via inhibiting palmitoyl-coenzyme A and L-serine condensation [35]. Results of replicate experiments showed that BSO at 1, 5, and 10 µM elicited significantly decreased (*p* < 0.05) nSMase activity by 18.75, 25, and 38.5%, respectively, compared to control cells lysate. In contrast, BSO at 0.1 and 0.5 µM decreased nSMase activity by 10.4 and 11.4%; respectively; however, this did not reach statistical significance. Treatment with 10 µM myriocin significantly decreased nSMase activity in HepG2 cell lysate by 40% compared to control. In contrast, 30 µM cambinol showed an insignificant (9%) decrease in HepG2 cell lysate nSMase activity compared to control cell lysate (Figure 4).

### 3.5. Effect of BSO, Cambinol and Myriocin on HepG2 Ceramide

**Figure 5:**
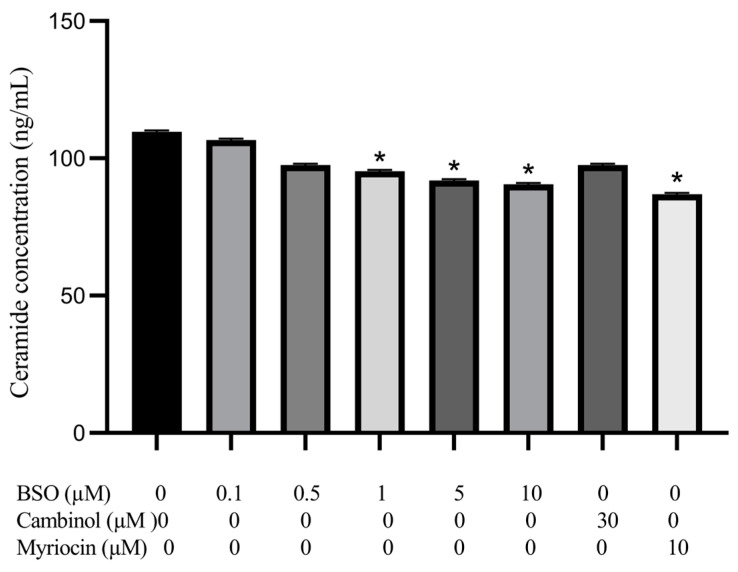
Effect of BSO, cambinol, and myriocin on HepG2 ceramide content. HepG2 cells were treated with BSO (0.1, 0.5, 1, 5, and 10 µM), cambinol (30 µM), and myriocin (10 µM) for 24 h. The ceramide content in cell lysates was determined using an enzyme-linked immunosorbent colorimetric assay. Data are expressed as the ceramide concentration (ng/mL) ± S.E. (n = 3), and statistically analyzed by *ANOVA* and *t*-test compared to control lysate. *, *p* < 0.05.

Figure 5 shows ceramide content in cell lysates following HepG2 cells treatment with BSO, cambinol, or myriocin. BSO at 1, 5, and 10 µM significantly (*p* < 0.05) reduced the ceramide content by 9, 11, and 12%, respectively, compared to control cell lysate, while BSO at 0.1 and 0.5 µM reduced the ceramide content by 2 and 7%, respectively. Treatment with 10 µM myriocin elicited a significant (*p* < 0.05) reduction of the ceramide content by 15% compared to control cell lysate. In contrast, treatment with 30 µM cambinol showed only a 7% non-significant decrease in ceramide content compared to the control cell lysate.

### 3.6. Effect of BSO on HepG2 Cell Proliferation

**Figure 6:**
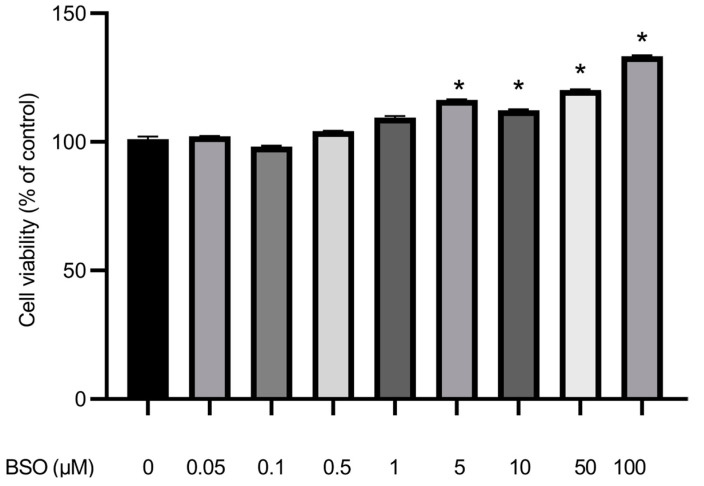
Effect BSO treatment on HepG2 cell proliferation. HepG2 cells were exposed to BSO (0.05, 0.1, 0.5, 1, 5, 10, 50, and 100 µM) for 24 h. Cell proliferation was measured using an MTT assay. Data are expressed as a percentage of control (at 100%) ± S.E. (n = 8) and statistically analyzed by ANOVA and *t*-test, compared to control cells (0 μM). *, *p* < 0.05.

Figure 6 shows that cell proliferation increased upon treatment with high BSO concentration. BSO at 0.1, 0.5, and 1 µM did not significantly affect tumor cell growth. Accordingly, 5, 10 µM, and higher BSO concentrations, 50 and 100 µM, had to be used to define BSO impact. The results indicated that almost total glutathione depletion significantly (*p* < 0.05) increased cell proliferation when compared to the cells still containing significant amounts of glutathione (Figure 6).

## 4. Discussion

Gamma-glutamyl-cysteinyl-glycine (glutathione) is a tripeptide α-amino acid that is an antioxidant [22,27,28,29]. In the reduced GSH and the oxidized GSSG, Glutatforms the principal intracellular antioxidant buffer against oxidative stress. BSO readily inhibits the biosynthesis of glutathione. The S-alkyl moiety of the sulfoximine of the BSO binds at the γ-GCL enzyme active site and hence, blocks the first enzyme used for glutathione biosynthesis (Supplementary Figure S2). Therefore, the synthesis of glutamyl cysteine is inhibited, so glutathione does not form [28,29,40,41].

The present work investigated the effects of BSO-mediated glutathione depletion in HepG2 cells. The obtained results fully confirmed the glutathione inhibitory action of BSO [22,28,29,40,41], which elicited a reduction of total glutathione content in HepG2 cell lysate from 20 to 15 μM upon the use of 0.1 and 0.5 μM, and to 2 μM following exposure to 5 or 10 μM BSO. Results also support the robust glutathione antioxidant activity [26,27,28,29], as its inhibition using as low as 0.1 μM BSO resulted in a dramatic increase in ROS levels.

Our results regarding the lack of nSMase2 mRNA detection in untreated HepG2 cells were in full accord with the findings of Revill et al. [11] and Karakashian et al. [12]. The inability to detect nSMase2 mRNA could be due to alterations or silencing of the encoding gene [9], or fast translation and trafficking of the protein product to the cell surface membrane, especially since nSMase activity could readily be demonstrated using cell surface membrane extracts of HepG2 and Huh-7 cells (data not shown). The results of this study are the first to show that BSO-mediated depletion of glutathione leads to a significant increase in nSMase1 and nSMase3 mRNA content. These results are encouraging, given the reported low nSMase1 and nSMase3 expression in HCC tissues, predicting poor long-term survival of HCC patients [14,15,16,17]. Despite eliciting an increase in nSMase1 and nSMase3 mRNA content and a dramatic increase in ROS levels, HepG2 glutathione depletion was associated with a significant (30%) decrease in cell lysate nSMase activity (Figure 4), partially supporting the findings, which document 70% inhibition of nSMase1 enzymatic activity by ROS and glutathione [15,19]. The limited (30%) HepG2 lysate nSMase activity reduction in the presence of tremendously high ROS levels might be attributed to the reversibility of the inhibition [15,19] or the presence of low amounts of nSMase2 protein hyperactivated by ROS [14,21,22,23,24,25]. In normal liver cells, the low nSMase2 activity can be activated during oxidative stress and glutathione depletion [22,23]. Simultaneous ROS-mediated nSMase1 inhibition and nSMase2 activation explain the consistently observed limited enzyme activity reduction. The absence of significant levels of nSMase2 protein is further supported by HepG2 cells treated in parallel with cambinol, a nSMase2-specific inhibitor [34], showing no significant change in cell lysate nSMase activity (Figure 4). A decrease in HepG2 lysate nSMase activity was achieved by the SM synthesis inhibitor, myriocin, known to reduce nSMases enzymatic activity, implying the involvement of other nSMases other than nSMase2 [42].

Reduced nSMase activity following BSO treatment was associated with a decrease in the content of the pro-apoptotic ceramide [7,8] in accord with impaired nSMase activity being reproducibly considered a major underlying mechanism for the reduced ceramide levels in HCC tissues [43]. The reductions in ceramide levels in lysates of BSO-treated HepG2 cells were, however, rather marginal (Figure 5). Consistent with this, tumor cells’ nSMase1 overexpression or knockdown failed to affect cell ceramide levels or viability [44,45]. Additionally, the limited changes in nSMases activity following BSO explains the marginal reductions in intracellular ceramide levels. The putative presence of nSMase2 protein, despite undetectable mRNA levels, is supported by ceramide levels restored to control cells following treatment with the nSMase2 inhibitor, cambinol [34].

Unimpaired content of the pro-apoptotic ceramide [7,8] in 0.05, 0.1, 0.5, and 1.0 μM BSO-treated HepG2 cells was associated with unaltered cell viability. Significant decrease in ceramide content in HepG2 cells treated with 5 and 10 μM BSO correlated with an increase in HepG2 cell proliferative potency. These findings are in full accord with Jin et al. [46] and Akhtar et al. [47], who reported that HepG2 cell viability was unscathed following treatment with BSO concentrations up to 200 μM. HepG2 cells survived treatment with one mM treatment with BSO for 20 h [48]. Conversely, in lung carcinoma A549 treated with BSO, the glutathione content dramatic decrease led to apoptosis due to an increase in ROS [49]. glutathione depletion and oxidative stress elicited Hela [50] and methocelioma [51] cell death. Liver and hepatoma cells renowned for their low nSMase2 content [9,10,11,12,13,14,15,16] and Figure 3 differ, as high BSO concentrations translated in extremely low glutathione content and tremendous increase in ROS accumulation led to a remarkable increase in HepG2 cell proliferative potential [46,47,48] and Figure 6.

The data support the role of high ROS concentrations in maintaining tumor cells’ vigorous proliferation rate [27,28,52,53] in some cancers and cell death signaling and execution in other cancer types [54,55].

## 5. Conclusions

The most salient outcome of this study is the lack of ready detection of nSMase2 mRNA in HepG2 cells before or after BSO treatment. Otherwise, HepG2 cell treatment with BSO did provoke a dramatic reduction in glutathione content and an increase in ROS levels, in addition to a considerable increase in nSMase1 and nSMase3 mRNA levels. However, almost complete inhibition of cell lysate nSMase activity and significantly lower ceramide levels were not observed, likely due to antagonistic effects on putative nSMase2 protein activity. Nevertheless, BSO treatment, especially at high doses, led to the potentiation of HepG2 cell proliferative capacity. The results indicate that the combined activity of nSMase1, nSMase2, and nSMase3 is required to control HepG2 cell proliferation. Extensive experiments are planned to define nSMase2 mRNA and protein content and activity in several cancer cell lines, biopsies, and normal para-cancer cells. After that, it is possible to examine whether changes in nSMase2 content predict the impact of glutathione depletion-mediated ROS levels on tumor cell growth or apoptosis [55] and determine whether glutathione depletion is a viable strategy for HCC management.

## Figures and Tables

**Table 1:** The sequence of forward (F) and reverse (R) primers used for the detection of neutral sphingomyelinases (nSMases) and glyceraldehyde 3-phosphate dehydrogenase (GAPDH) mRNA detection.

Primer	Sequence 5′ to 3′
nSMase1-F	TTTGGTGTCCGCATTGACTA
nSMase1-R	TAGAGCTGGGGTTCTGCTGT
nSMase2-F	GGAAGGCCGAGGTGGAA
nSMase2-R	CCCCCGAAGACACCATCA
nSMase3-F	CACCCAGGATGAGAATGGAAA
nSMase3-R	GTCCGTCCTCACCCACGAT
GAPDH-F	AGCCACATCGCTCAGACAC
GAPDH-R	GCCCAATACGACCAAATCC

## Data Availability

The data presented in this study are available in the article and supplementary materials.

## Supplementary Materials

The following supporting information can be downloaded at: https://www.mdpi.com/article/10.3390/cimb45060318/s1, Figure S1: Surface membrane sphingomyelin is hydrolyzed to phosphocholine and ceramide by membrane-associated neutral sphingomyelinase 2 (nSMase2), which is activated by oxidative stress and glutathione (GSH) depletion; Figure S2: Buthionine sulfoximine (BSO) inhibits the formation of glutathione (GSH) and thus reduces cellular antioxidant capacity.
