# Supplementary material for: Impact of HepG2 Cells Glutathione Depletion on Neutral Sphingomyelinases mRNA Levels and Activity
Source: Curr Issues Mol Biol. 2023 Jun 8;45(6):5005–17. doi: 10.3390/cimb45060318 (PMC10296953; doi:10.3390/cimb45060318)
Supplement: Supplementary file 1 [file cimb-45-00318-s001.zip › Supplementary Figure S1.pdf]

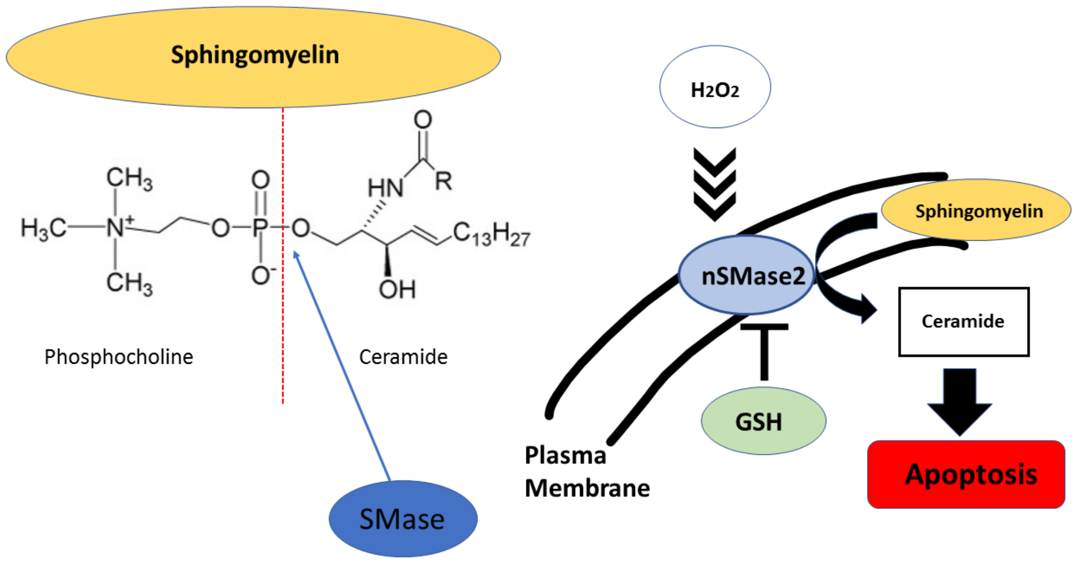

Supplementary Figure S1. Surface membrane sphingomyelin is hydrolyzed to phosphocholine and ceramide by membrane-associated neutral sphingomyelinase 2 (nSMase2), which is activated by oxidative stress and glutathione (GSH) depletion.
